# Syndrome du canal carpien secondaire à une variété anatomique rare du nerf médian

**DOI:** 10.11604/pamj.2018.31.39.15526

**Published:** 2018-09-20

**Authors:** Amine Tahir, Amine Sdoudi, Mohamed Chahed, Aniss Elbaitil, Lkoutbi Fakherdine, Yasser Sbihi, Driss Bennouna, Mustafa Fadili

**Affiliations:** 1 Traumatologie-Orthopédie Aile 4, CHU Ibn Rochd, Casablanca, Maroc

**Keywords:** Carpal tunnel syndrome, anatomical variety of the median nerve, neurolysis, Carpal tunnel syndrome, anatomical variety of the median nerve, neurolysis

## Abstract

Le syndrome du canal carpien représente l'un des motifs de consultation les plus fréquents en chirurgie de la main, son incidence annuelle est de 300 par 100.000 habitants et 80.000 interventions chirurgicales pour le syndrome du canal carpien sont réalisées chaque année en France. Dans la plupart des cas, le syndrome du canal carpien est idiopathique survenant sans qu'aucune cause puisse être identifiée, cependant en dehors de ces formes l'intrication de plusieurs mécanismes peuvent expliquer la relation cause à effet de cette pathologie. A travers cette observation nous allons essayer de mettre la lumière sur une association exceptionnelle d'un syndrome du canal carpien secondaire à une variété anatomique rare du nerf médian.

## Abstract (en)

Carpal tunnel syndrome is one of the the most common reasons for consultation in hand surgery with an annual incidence of 300 per 100 000 inhabitants and, in France, of 80 000 surgical interventions. In most cases, carpal tunnel syndrome is idiopathic occurring without a recognizable cause. However, beyond these forms, the merging of several mechanisms can explain the cause-and-effect relationship of this pathology . This study aimed to highlight an exceptional association of a carpal tunnel syndrome secondary to a rare anatomical variety of the median nerve.

## Introduction

Le syndrome du canal carpien est l'ensemble des manifestations cliniques et électriques secondaires à la compression ou à l´irritation du nerf médian dans le canal carpien. Ce dernier étant un espace ostéofibreux inextensible situé à la face antérieure du carpe. La première description clinique fut décrite par Paget en 1854, cependant la corrélation anatomocliniqueest attribuée à Marie en 1913 [1-3]. Il est le plus fréquent des syndromes canalaires du membre supérieur, l'origine idiopathique n'est retenue qu'après avoir éliminé les causes endocriniennes, post traumatiques, iatrogènes, rhumatismale et anatomiques [1]. La recherche systématique d´une étiologie est rarement couronnée de succès, dans notre cas il s'agit d'une étiologie très rare fortuitement découverte. Il s'agit notamment d'une variété anatomique du nerf médian jamais décrite dans la littérature médicale.

## Patient et observation

**Figure 1:**
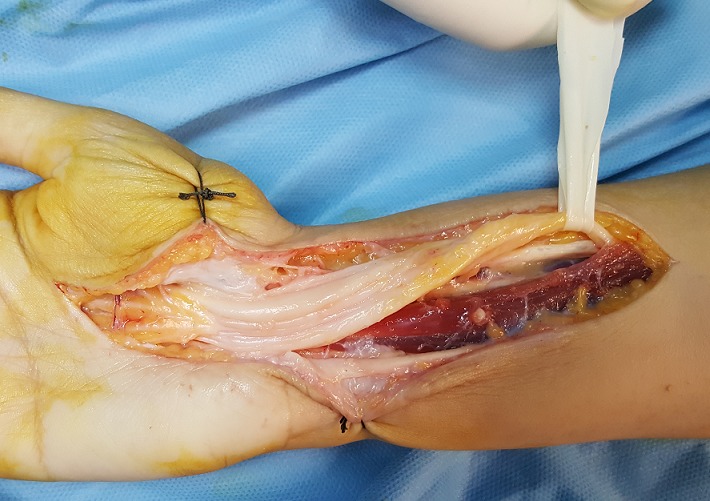
Aspect multi fasciculaire du nerf median

**Figure 2:**
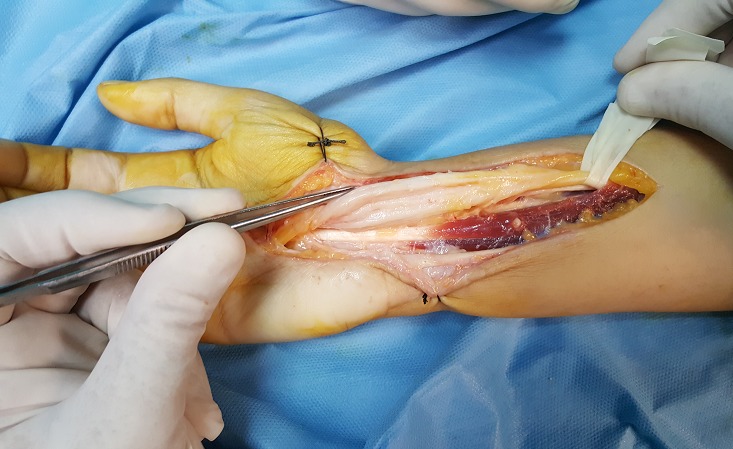
Aspect blanc nacré du nerf médian au niveau du canal carpien

**Figure 3:**
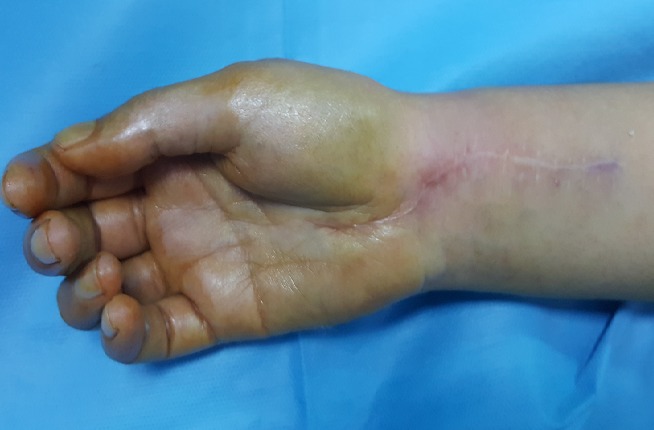
Aspect général après un an avec une trophicité normale de la loge thénar

Il s'agit d'une observation d'une patiente âgée de 26 ans, institutrice de profession, mariée, mère d'un enfant, droitière de la latéralité qui avait consulté pour des paresthésies doigts nocturnes et à l'effort manuel au niveau des trois premiers doigts. Nous avons noté une faiblesse de la préhension obligeant la patiente à arrêter les gestes quotidiens. L'examen clinique avait objectivé à l'inspection une peau sèche et luisante au niveau du territoire sensitif du nerf médian de la main témoignant des troubles trophiques secondaires ainsi qu'une hypotrophie de la loge thénar. Le testing de la force musculaire des muscles de la loge thénar était respectivement de l'ordre de 3 pour le muscle court abducteur, de 2 pour l'opposant et 3 pour le court fléchisseur du pouce. Les tests de provocation étaient positifs: le test de Tinel réveille la douleur au niveau du territoire du nerf médian en percutant le rétinaculum des fléchisseurs, par ailleurs, le test de Phalen en flexion du poignet maintenue pendant une minute accentue la douleur. L'examen de la sensibilité avait objectivé une anesthésie du premier deuxième et la moitié externe de l'annulaire au niveau de face palmaire, ainsi que la face dorsale des 2 dernières phalanges de l'index et du major. Le bilan radiologique a consisté en une radiographie standard qui n'a révélé aucune anomalie osseuse pouvant expliquer la symptomatologie de la patiente. L'EMG avait montré une réduction des vitesses de conductions motrices et sensitives du nerf médian. Nous avons procédé à une libération du nerf médian par aponévrotomie du rétinaculum des fléchisseurs à ciel ouvert, qui a permis de visualiser le nerf médian et nous avons constaté la variété multi fasciculaire (minimum 7 contingents) avec aspect aplati blanc nacré du nerf (Figure 1, Figure 2). On a réalisé une dissection après élargissement en proximal et en distal selon la voie de Henry et nous avons conclu que l'aspect du nerf ne correspondait pas à la normale mais une cause tumorale a été éliminée. Les suites opératoires furent simples, La prise en charge a été compléter par 20 séances de rééducation avec une bonne récupération du score de la force musculaire et disparition des troubles trophiques. Après un recul de 1 an nous n'avons pas noté de récidive (Figure 3).

## Discussion

**Figure 4:**
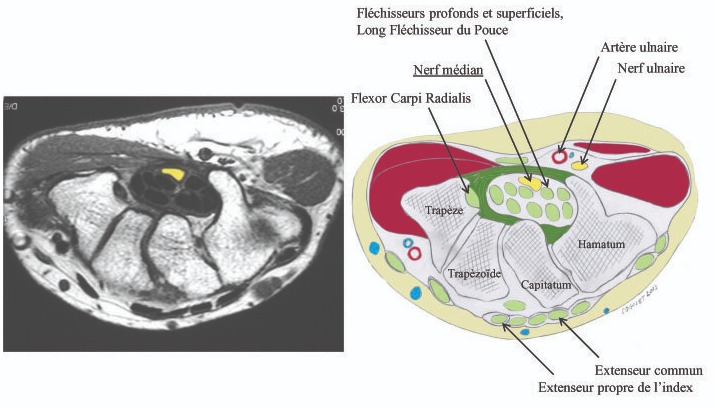
Position anatomique du nerf médian au niveau du canal carpien

Le syndrome du canal carpien est la pathologie canalaire prédominante par excellence et qui correspond à l'irritation du nerf médian dans sa traversée du canal carpien. Il touche 1% de la population, son incidence annuelle varie selon les pays, en France il est évalué à 80/100 000 habitants [5-7]. On note une prédominance féminine avec un sexe ratio 3/1, la tranche d'âge la plus touchée est de 40 à 60 ans, il peut être bilatéral dans 50% des cas, et lorsqu'il est unilatéral il touche surtout le côté prédominent dans 2/3 des cas [3, 4]. Le passage du nerf médian dans ce canal étroit et inextensible, qui est fermé en avant par le rétinaculum des fléchisseurs, rend toute augmentation de pression à l'intérieur susceptible de retentir sur le nerf médian. L'augmentation de la pression intra canalaire peut avoir diverses causes, parfois elle est d'origine multifactorielle [8-10] (Figure 4). La variété anatomique particulière du nerf médian comme étiologie du syndrome du canal carpien n'est pas décrite dans la littérature, de ce fait la comparaison de notre cas avec un cas similaire dans la littérature s'avère difficile, néanmoins l'origine tumorale au dépend du nerf médian a été écarté en peropératoire vu que son aspect ne correspondait pas au caractère reconnu anatomo-clinique des tumeurs. Dans une étude qui a été porté sur 98 cadavres soit 196 membres supérieurs a objectivé une variation de formation du nerf médian au niveau du plexus brachial et du bras par 3 racines ainsi que des anastomoses avec le nerf musculo cutané et le nerf ulnaire, cependant cette étude n'a pas cité une variation de forme au niveau du poignet, ce qui prouve la rareté de notre cas [11]. L'aspect multifascicullaire du nerf médian au niveau du canal carpien qui est un espace clos augmente la pression intra canalaire, ce qui va retentir sur le nerf lui-même, en se traduisant cliniquement par un syndrome canalaire, d'autant plus que la bonne évolution clinique après sa libération explique en partie la responsabilité de cette variété dans l'apparition du syndrome du canal carpien chez notre patiente. Pour notre patiente, l'intervention chirurgicale a consisté en une libération du nerf en sectionnant le rétinaculum des fléchisseurs ainsi que la recherche d'une étiologie pouvant expliquer la compression du nerf. La variante anatomique multifascicullaire du nerf médian augmente la pression au niveau du canal carpien, ce qui n'est pas tolérable par le nerf. Le cas de notre patiente associe cette variante inhabituelle du nerf à l'exposition professionnelle ce qui a rapidement fait exprimé la symptomatologie en âge jeune de 30 ans.

## Conclusion

Le syndrome du canal carpien est très fréquent dans notre contexte, la variante anatomique inhabituelle du nerf est rarement évoquée vu sa rareté, et aucune série dans la littérature ne permet de faire une comparaison de résultat avec notre cas, la libération du nerf que ce soit ciel ouvert ou par voie arthroscopique représente la seule solution d'arrêter l'évolution vers une amyotrophie dégénérative des muscles permettant ainsi la reprise de la fonction normale de la main.

## Conflits d’intérêts

Les auteurs ne déclarent aucun conflit d'intérêt.

## Contributions des auteurs

Tous les auteurs ont contribué à la redaction de ce travail. Les auteurs déclarent avoir lu et approuvé la version finale de ce travail.
